# Tackling aging muscle loss throughout lesser mealworm protein supplementation

**DOI:** 10.1016/j.jnha.2024.100407

**Published:** 2024-11-11

**Authors:** Bruno Remigio Cavalcante, Mariana Ferreira de Souza

**Affiliations:** a Department of Physical Education, Clinical Exercise Laboratory (LABEC), Universidade Federal do Vale do São Francisco (UNIVASF), Petrolina, PE, Brazil; b Graduate Program in Physical Education (PPGEF), Universidade Federal do Vale do São Francisco (UNIVASF), Petrolina, PE, Brazil; c Graduate Program in Rehabilitation and Funcional Performance (PPGRDF), Universidade de Pernambuco (UPE), Petrolina, PE, Brazil

The dynamic process of muscle protein synthesis (MPS) and muscle protein breakdown (MPB) determine whether the net balance between MPS/MPB will result in muscle mass gain (positive balance), atrophy (negative balance) or maintenance [1]. As we age, the skeletal muscle commonly faces a physiological phenomenon describe as anabolic resistance, which is characterized by reduced sensitive to anabolic stimuli such as exercise and nutrition [1,2]. The age-related loss of muscle mass (e.g., sarcopenia) begins in our 4–5th decade of life, with estimates suggesting that muscle mass deterioration occurs at a rate ranging from 0.8 to 1% per year [3].

Epidemiological studies [[4], [5], [6], [7], [8]] also shown that skeletal muscle mass (SMM) decline/sarcopenia are marked associated with risk of developing many chronic diseases and several adverse outcomes, including cardiovascular diseases, type 2 diabetes, cancer, and brain and mental health issues, falls, challenge recovery from major injuries, bone fractures, hospitalizations, and premature death. Thus, preserving SMM is crucial for healthy aging [5], as it is linked to functional abilities/locomotion—such as the ability to move around, get up from a chair, and transfer— force production, metabolic regulation as well as physical independence and health-related quality of life.

Protein-based nutrition along with exercise training (e.g., resistance training) are core interventions capable of driving cellular and molecular signals to overcome the anabolic resistance and improve SMM in the context of aging. According to The Food and Nutrition Board, a recommended dietary allowance (RDA) of 0.8−0.9 g/kg/day of protein is needed for most adults ≥18 years old [9]. For healthy older individuals and those with malnourished or at risk of malnutrition, the optimal level of protein intake values varies between 1.0–1.2 and 1.2–1.5 g/kg/day, respectively [10]. High-quality protein sources can be achieved through foods such as lean meats, fish, eggs, dairy products, and grains. Animal- and plant-based protein supplements may also provide the ideal quantity/quality of nutrients to promote muscle health [11]. Systematic reviews [[12], [13], [14]] support the notion that protein supplementation is effective to improve lean body mass in older individuals (Fig. 1).

**Fig. 1:**
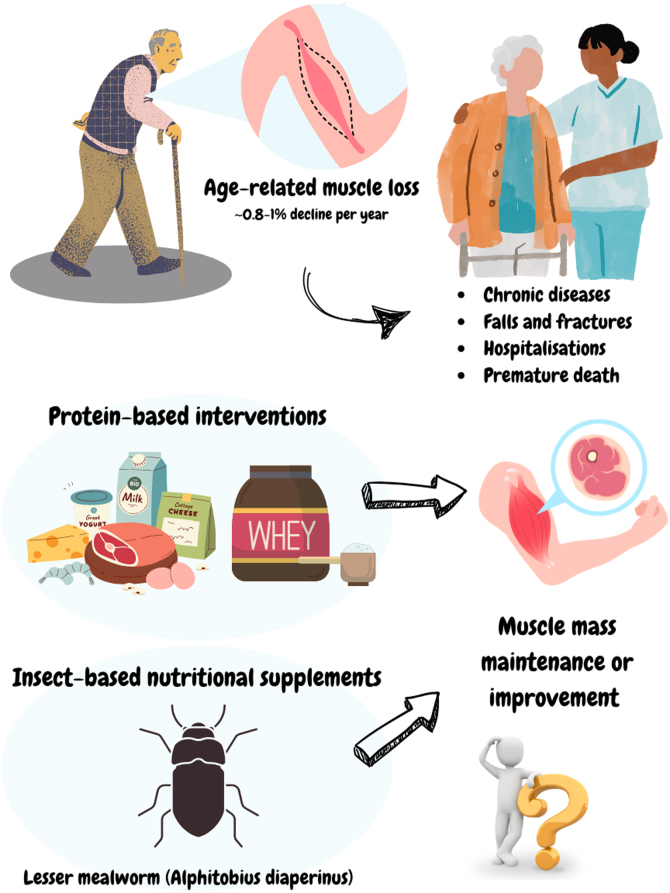
Impact of age-related muscle loss and the role of protein-based interventions to promote muscle health. (Created with canva.com).

Over the past few years, insect-based supplementation has garnered attention as a viable alternative to milk-based proteins (Fig. 1), particularly because of its well-balanced amino acid composition that meets essential amino acid requirements [[15], [16], [17]]. In this issue of The Journal of Nutrition, Health and Aging (JNHA), Koopmans and colleagues [18] present noteworthy findings from a randomized controlled trial investigating the effects of 11 weeks of daily supplementation with lesser mealworm protein (LMP) on SMM in healthy older adults. A total of 59 participants were randomly assigned to receive LMP, whey protein (WHEY), or a placebo. The outcomes measured included total SMM, fat mass assessed by bioelectrical impedance, anthropometry, leg strength, and habitual physical activity levels. The results indicated that the LMP group experienced greater gains in SMM compared to both the WHEY and placebo groups. Additionally, both the LMP and WHEY groups showed a decrease in fat mass and handgrip strength throughout the intervention. However, the authors did not observe substantial changes in normalized one-repetition maximum (1RM) leg muscle strength. Based on these findings, authors highlighted the potential of insect-based proteins to target age-related muscle loss in older adults with an active lifestyle behavior.

Despite the novelty findings, several gaps remain unclear. Further randomized controlled trials with larger samples are desirable to confirm the efficacy of LMP on SMM improvements and whether these changes are clinically pertinent compared with placebo and standard care interventions. These trials need to incorporate precise methods to accurately examine changes in muscle mass in response to nutrition strategies (e.g., DXA, CT-, MRI-based methods, and others). In terms of eligible criteria, researchers also should consider include older participants with sarcopenia/frailty markers (e.g., low levels of physical activity, higher sedentary behavior, mobility limitations) in order to understand the potential of insect-based protein interventions to mitigate age-related muscle mass loss within clinical populations. Another gap that needs to be addressed is whether the effects of LMP could be optimized when combined with exercise training, such as resistance training. Lastly, as previously mentioned by the authors, future research should adjust the dosage of the protein according to the participant body weight to avoid over- and underdose of the supplement.

In conclusion, the article by Koopmans and colleagues [18] demonstrates that insect-based protein supplementation, particularly lesser mealworm protein (LMP), represents a promising intervention for preserving or enhancing age-related changes in muscle mass. These findings have significant practical implications considering the limited availability of sufficient, high-quality dietary protein sources. Future works are critically necessary to elucidate the gaps and issues previously highlighted to shed light in this field of research.

## Declaration of competing interest

We do not have conflict of interest to disclose.

## References

[1] Wilkinson D.J., Piasecki M., Atherton P.J. (2018). The age-related loss of skeletal muscle mass and function: measurement and physiology of muscle fibre atrophy and muscle fibre loss in humans. Ageing Res Rev..

[2] Morton R.W., Traylor D.A., Weijs P.J.M., Phillips S.M. (2018). Defining anabolic resistance: implications for delivery of clinical care nutrition. Curr Opin Criti Care..

[3] Goodpaster B.H., Park S.W., Harris T.B., Kritchevsky S.B., Nevitt M., Schwartz A.V. (2006). The loss of skeletal muscle strength, mass, and quality in older adults: the health, aging and body composition study. J Gerontol A Biol Sci Med Sci..

[4] Cruz-Jentoft A.J., Sayer A.A. (2019). Sarcopenia. Lancet..

[5] Sayer A.A., Cooper R., Arai H., Cawthon P.M., Ntsama Essomba M.-J., Fielding R.A. (2024). Sarcopenia. Nat Rev Dis Primers..

[6] Uchida K., Sugimoto T., Tange C., Nishita Y., Shimokata H., Saji N. (2023). Association between Reduction of muscle mass and faster declines in global cognition among older people: a 4-year prospective cohort study. J Nutr Health Aging..

[7] Xue T., Gu Y., Xu H., Chen Y. (2024). Relationships between sarcopenia, depressive symptoms, and the risk of cardiovascular disease in Chinese population. J Nutr Health Aging..

[8] Zhou H., Ding X., Luo M. (2024). The association between sarcopenia and functional disability in older adults. J Nutr Health Aging..

[9] Phillips S.M., Paddon-Jones D., Layman D.K. (2020). Optimizing adult protein intake during catabolic health conditions. Adv Nutr..

[10] Deutz N.E., Bauer J.M., Barazzoni R., Biolo G., Boirie Y., Bosy-Westphal A. (2014). Protein intake and exercise for optimal muscle function with aging: recommendations from the ESPEN Expert Group. Clin Nutr..

[11] Devries M.C., Phillips S.M. (2015). Supplemental protein in support of muscle mass and health: advantage whey. J Food Sci..

[12] Wirth J., Hillesheim E., Brennan L. (2020). The role of protein intake and its timing on body composition and muscle function in healthy adults: a systematic review and meta-analysis of randomized controlled trials. J Nutr..

[13] Morton R.W., Murphy K.T., McKellar S.R., Schoenfeld B.J., Henselmans M., Helms E. (2018). A systematic review, meta-analysis and meta-regression of the effect of protein supplementation on resistance training-induced gains in muscle mass and strength in healthy adults. Br J Sports Med..

[14] Li M.-L., Zhang F., Luo H.-Y., Quan Z.-W., Wang Y.-F., Huang L.-T. (2024). Improving sarcopenia in older adults: a systematic review and meta-analysis of randomized controlled trials of whey protein supplementation with or without resistance training. J Nutr Health Aging..

[15] Hermans W.J.H., Senden J.M., Churchward-Venne T.A., Paulussen K.J.M., Fuchs C.J., Smeets J.S.J. (2021). Insects are a viable protein source for human consumption: from insect protein digestion to postprandial muscle protein synthesis in vivo in humans: a double-blind randomized trial. Am J Clin Nutr..

[16] Vangsoe M.T., Thogersen R., Bertram H.C., Heckmann L.-H.L., Hansen M. (2018). Ingestion of insect protein isolate enhances blood amino acid concentrations similar to soy protein in a human trial. Nutrients..

[17] Koopmans L., Spoelder M., Bongers C.C.W.G., Eijsvogels T.M.H., Hopman M.T.E. (2024). The effect of lesser mealworm protein on exercise-induced muscle damage in active older adults: a randomized controlled trial. J Nutr Health Aging..

[18] Koopmans L., Spoelder M., Bongers C., Eijsvogels T.M.H., Hopman M.T.E. (2024). Daily supplementation of lesser mealworm protein for 11-weeks increases skeletal muscle mass in physically active older adults. J Nutr Health Aging..

